# High-salt diet does not boost neuroinflammation and neurodegeneration in a model of α-synucleinopathy

**DOI:** 10.1186/s12974-020-1714-y

**Published:** 2020-01-24

**Authors:** Antonio Heras-Garvin, Violetta Refolo, Markus Reindl, Gregor K. Wenning, Nadia Stefanova

**Affiliations:** 1 Department of Neurology, Division of Neurobiology, Medical University of Innsbruck, Innrain 66, 6020 Innsbruck, Austria; 2 Department of Neurology, Neuroimmunology Research Group, Medical University of Innsbruck, Innrain 66, 6020 Innsbruck, Austria

**Keywords:** Neuroinflammation, High-salt diet, α-Synuclein, Multiple system atrophy, Parkinson’s disease

## Abstract

**Aim:**

Pre-clinical studies in models of multiple sclerosis and other inflammatory disorders suggest that high-salt diet may induce activation of the immune system and potentiate inflammation. However, high-salt diet constitutes a common non-pharmacological intervention to treat autonomic problems in synucleinopathies such as Parkinson’s disease and multiple system atrophy. Since neuroinflammation plays an important pathogenic role in these neurodegenerative disorders, we asked here whether high-salt diet may aggravate the disease phenotype in a transgenic model of multiple system atrophy.

**Methods:**

Nine-month-old PLP-hαSyn and matched wildtype mice received normal or high-salt diet for a period of 3 months. Behavioral, histological, and molecular analyses were performed to evaluate the effect of high-salt diet on motor decline, neuroinflammation, neurodegeneration, and α-synuclein accumulation in these mice.

**Results:**

Brain subregion-specific molecular and histological analyses showed no deleterious effects of high-salt diet on the level of microglial activation. Moreover, neuroinflammation-related cytokines and chemokines, T cell recruitment or astrogliosis were unaffected by high-salt diet exposure. Behavioral testing showed no effect of diet on motor decline. High-salt diet was not related to the deterioration of neurodegeneration or α-synuclein accumulation in PLP-hαSyn mice.

**Conclusions:**

Here, we demonstrate that high-salt diet does not aggravate neuroinflammation and neurodegeneration in PLP-hαSyn mice. Our findings discard a deleterious pro-neuroinflammatory effect of high-salt diet in multiple system atrophy.

## Introduction

High-salt diet (HSD) has been associated with chronic inflammation [1], neuroinflammation [2, 3], and autoimmune diseases [4, 5]. In this regard, high-salt intake has been shown to affect immune cells and induce the differentiation of T helper (Th)17 cells with pathogenic phenotype [6, 7] which play an important role in the induction of neuroinflammation, CNS autoimmunity, and neurovascular and cognitive dysfunction [8–11]. The induction of pathogenic Th17 cells and their infiltration in the CNS by HSD has shown to potentiate neuroinflammation in experimental autoimmune encephalomyelitis (EAE), an animal model which mimics many features of multiple sclerosis (MS) [6, 9, 12, 13]. In this mouse model, HSD accelerates disease onset, augments its severity, and enhances blood-brain barrier (BBB) disruption and brain pathology [6, 7, 14, 15]. Moreover, in experimental models high salt also promotes pro-inflammatory phenotype of myeloid cells by enhancing cytokine production and polarization towards M1 phenotype, leading to an overall imbalance of immune homeostasis [14, 16, 17]. In accordance with these findings, a recent study demonstrated that increased sodium intake is associated with clinical disease exacerbation, augmented relapse risk, and increased magnetic resonance imaging (MRI) activity in MS patients [4]. However, larger clinical studies recently failed to show an association between salt intake and higher MS disease risk, progression, or activity [18, 19], thus the possible deleterious effect of HSD in MS is still under discussion.

Synucleinopathies such as Parkinson’s disease (PD) and multiple system atrophy (MSA) constitute neurodegenerative disorders characterized by autonomic failure, motor impairment and the abnormal accumulation of α-synuclein (α-syn) in the cytoplasm of either neurons (Lewy bodies, characteristics of PD) or in oligodendroglial cytoplasmic inclusions (GCIs, characteristics of MSA) [20–23]. The accumulation of α-syn in MSA brains is associated with microglial activation and neuroinflammation [24–26], demyelination [27, 28], and neurodegeneration [29]. MSA is subdivided into two motor subtypes depending on the main brain areas affected by the pathology, the parkinsonian variant (MSA-P) characterized by striatonigral degeneration (SND) and the cerebellar variant (MSA-C) characterized by olivopontocerebellar atrophy (OPCA), but autonomic failure is present in both variants [30–32]. At present, there are no disease-modifying therapies to stop disease progression and only mitigation of some symptoms is feasible [33]. In this regard, increased fluid and salt intake is often recommended by physicians to alleviate neurogenic orthostatic hypotension [34].

The association of HSD with pro-inflammatory disorders together with the experimental evidence showing its deleterious effects in different in vitro and in vivo models questions the use of HSD in MSA and PD patients. The fact that neuroinflammation and the immune system, especially microglial cells, play an important role in MSA and PD pathogenesis [24–26, 35–37] and the recent evidences of a possible involvement of Th17 cells in PD [38–43] support these concerns. In order to evaluate the pathological consequences of HSD in α-synucleinopathies we have analyzed its effects in the PLP-hαSyn transgenic mice model of MSA. Here, we show that high dietary intake of salt does not accelerate disease progression nor increase neuroinflammation, microglial activation, or neurodegeneration in MSA mice, suggesting that HSD may not have a pro-neuroinflammatory effect in this particular α-synucleinopathy.

## Material and methods

### Animals and treatments

Clinical and pathological features of MSA are recapitulated in the PLP-hαSyn mouse model. These mice overexpress human wildtype α-syn in oligodendrocytes under the myelin proteolipid protein (PLP) promoter [44] leading to the formation of GCI-like structures, autonomic failure, progressive SND, and motor impairment [44–54]. SND in the PLP-hαSyn mice is characterized by a reduction in the number of dopaminergic neurons in the substantia nigra pars compacta (SNc) [55] followed by a reduction of the density of dopaminergic terminals and medium spiny neurons (MSNs) in the striatum linked to significant motor decline [51]. Similar to the human pathology, PLP-hαSyn mice develop progressive microglial activation initially triggered by α-syn pathology [51, 53]. Our group has also previously shown that stressors relevant to human MSA, e.g., mitochondrial dysfunction [52] or proteolysis disruption [56], can aggravate the pathology in PLP-hαSyn mice towards full-blown MSA with spreading of GCI, SND, OPCA, and strong microglial activation. In summary, the PLP-hαSyn mouse model provides an important and relevant pre-clinical tool to study disease mechanisms of MSA progression downstream of the accumulation of α-syn in oligodendrocytes.

PLP-hαSyn and C57BL/6 N wildtype animals were kept under temperature-controlled pathogen-free conditions on a light/dark 12 h cycle. Nine-month-old PLP-hαSyn and wildtype animals were both randomized in 2 groups, one fed with normal food pellets (0.19% sodium; SSNIFF Spezialdiäten GmbH) and tap water, another fed with HSD (4% NaCl; SSNIFF Spezialdiäten GmbH) and tap water containing 1% NaCl as previously described [2, 6, 7]. After 3 months of treatment, the animals were sacrificed and brains were collected. Bodyweight of all animals was measured weekly throughout the treatment period (Additional file 1: Figure S1). Although PLP-hαSyn mice presented lower body weight compared to healthy control animals, no differences due to diet were observed within the 2 animal groups (Additional file 2: Figure S1). All the experiments were performed according to the ethical guidelines of the EU (Directive 2010/63/EU for animal experiments) and the Austrian Federal Ministry of Science and Research (permission BMFWF-66.011/0018-WF/v/3b/2015). All analyses were done by a researcher who was blinded to the treatment of the animals.

### Stride length analysis

Stride length analysis was performed with DigiGait™ Imaging System (Mouse Specifics Inc.) as previously described [51, 56]. Briefly, mice were placed onto a transparent treadmill belt and gait of each mouse was recorded with a video camera placed below the belt. Stride length was analyzed with DigiGait Software 9.0 (Mouse Specific, USA) and expressed in cm.

### Tissue processing and histology

Animals were perfused intracardially with phosphate-buffered saline (PBS, pH 7.4, Sigma) under deep thiopental anesthesia and brains were extracted. Hemibrains were post-fixed overnight in 4% paraformaldehyde (pH 7.4, Sigma) at 4 °C and then cryoprotected in 30% sucrose (in PBS). Finally, the brains were frozen using 2-Bodyweight (Sigma) and stored at − 80 °C. The brains were cut in 40 μm thick coronal sections using a freezing microtome (Leica) and stored free-floating in a cryoprotectant buffer at − 20 °C.

### Immunohistological analyses

Free-floating sections were immunostained following standard protocols. Microglial activation was evaluated by immunofluorescence using antibodies against IBA1 (1:600, WAKO) and CD68 (1:200, R&D). In order to evaluate the level of SND, SNc sections were stained with anti-tyrosine hydroxylase (TH) antibody (1:1000, Millipore) and the number of dopaminergic (TH+) neurons was analyzed by stereological counting. Striatal sections were stained with anti-DARPP32 antibody (BD Bioscience; 1:2000) and the number medium spiny neurons (MSNs; DARPP32+) was quantified. OPCA in the cerebellum was evaluated by DARPP32 immunostaining of Purkinje cells (DARPP32+). To analyze the number of GCIs, representative sections including SNc, cerebellar white matter (CBWM), and motor cortex (M2) were stained with anti-phosphorylated α-syn antibody (pS129; 1:1000, Abcam). For immunofluorescence, suitable secondary anti-IgG antibodies conjugated with Alexa 488 or Alexa 594 (Life Technologies) were applied and coverslipped with mounting medium Fluromount-G (Southern Biotech). For immunohistochemistry, sections were incubated with biotinylated secondary antibodies followed by Vectastain ABC reagent (Vector Laboratories) and 3,3′-diaminobenzidine (Sigma) to visualize the binding sites. Stained sections were mounted on slides, dehydrated, and coverslipped with Entellan (Merck).

### Image analyses

Neuroanatomy was assessed using a Mouse Brain Atlas. For microglial activation assessment, images were acquired with a fluorescence microscope (Leica DMI4000) and the positive area for IBA1 or CD68 was estimated using ImageJ (National Institutes of Health). Results are presented as percentage of IBA1 or CD68 area per section total area. Stereological analysis was performed using the Nikon E-800 microscope equipped with Nikon digital camera DXM1200 and Stereoinvestigator software (Microbrightfield Europe e.K) as described previously [56]. The number of TH+ neurons in the SNc and DARPP-32+ neurons in the *striatum* was measured by applying the optical fractionator workflow [51]. The density of GCIs and Purkinje cells (DARPP32+) were assessed with meander scan and is expressed in GCI/mm^2^ and DARPP32+ neurons/mm^2^ respectively.

### RNA extraction and quantitative RT-qPCR

For molecular analyses, hemibrains were quickly dissected in the forebrain, midbrain, cerebellum, and brainstem, frozen in liquid nitrogen and stored at − 80 °C. RNA was extracted using TRIzol reagent (Life technologies) according to the manufacturer’s instructions. Tissue was homogenized with ULTRA-TURRAX T-8 basic tissueruptor (IKA) in the presence of TRIzol. RNA samples (3 μg) were retrotranscribed to cDNA using High-Capacity cDNA Reverse Transcription Kit (Applied-Biosystems). Real time PCR was performed in a 7500 Real-Time PCR Systems (Applied-Biosystems) using TaqMan™ Universal PCR Master Mix (Applied-Biosystems). *Gapdh* mRNA levels were estimated to normalize for mRNA input amounts. TaqMan probe sequences are available upon request. mRNA levels were obtained using the 2^−ΔΔCt^ method and expressed as fold-change relative to the wildtype normal diet control group [57].

### Cytokine/chemokine levels

Fresh frozen forebrain, midbrain, cerebellum, and brainstem were homogenized in Triton-X (TX) extraction buffer (50 mM Tris-base pH 7.6, 150 mM NaCl, 1% Triton-X-100, 2 mM EDTA) containing protease and phosphatase inhibitors. The lysates were centrifuged (16,000×*g* for 10 min at 4 °C) to remove debris and the supernatant was collected and stored at − 80 °C. Protein concentrations were determined with BCA Protein Assay Kit (Sigma). ProcartaPlex® Multiplex Immunoassay system (eBioscience, Waltham, MA USA) was used to simultaneously measure the concentration of different cytokines and chemokines. The same protein amount was loaded for all samples. Duplicates were performed per each sample and mean values were calculated for subsequent statistical analysis. Data are presented as pg cytokine/chemokine per mg total protein.

### Dot blot analysis of soluble α-syn

Lysates obtained previously were ultra-centrifuged (100,000×*g* for 60 min at 4 °C) and the supernatant was collected and stored at − 80 °C. Equal amounts of protein (5 μg) per sample were spotted onto nitrocellulose membranes (GE Healthcare) and air-dried for 30 min. Membranes were incubated overnight at 4 °C in blocking buffer (PBS, pH 7.6, 0.1% Tween 20, 5% non-fat dry milk) with primary antibody against human α-syn (4B12; 1:1000, Genetex). Signal detection was performed using HRP-conjugated secondary antibodies and WesternBright Quantum kit (Advansta). Images were acquired using the Fusion FX system for western blot and gel imaging and quantified with FUSION CAPT V16.09b software (Vilber Lourmat).

### Statistical analyses

All statistical analyses were conducted using the software Graph-Pad Prism 7 (Graphpad Software). The mean ± S.E.M was used to present the results. Two-way analysis of variance (ANOVA) with post hoc Bonferroni test was used to compare the groups if not indicated otherwise. A *p* value < 0.05 was considered statistically significant.

## Results

### High-salt diet causes partial upregulation of genes linked to microglial and astroglial activation without changes at protein level in PLP-hαSyn brains

**Fig. 1:**
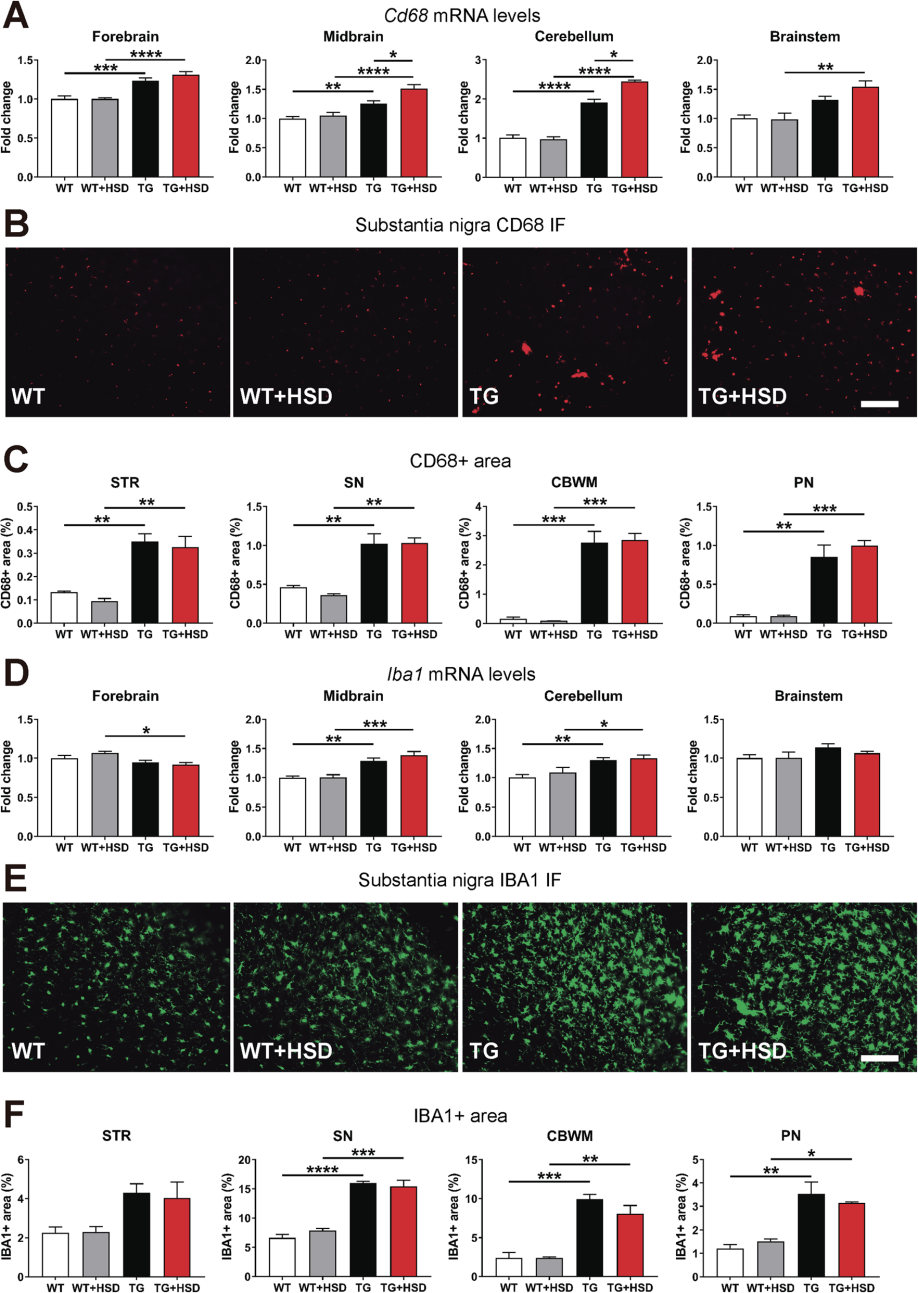
High-salt diet does not affect microglial activation in MSA mice. **a** Relative gene expression (mRNA levels) of the microglial activation marker *Cd68* in different brain regions. The data is expressed in fold change relative to WT mice fed with a normal diet. **b** Representative immunofluorescence (IF) images of the substantia nigra (SN) sections stained against CD68 (in red). Scale bar, 150 μm. **c** CD68 positive (CD68+) area in different brain regions (STR: striatum; SN; CBWM: cerebellar white matter; PN: Pontine nuclei) determined by ImageJ analysis and expressed as the % of the total area. **d**, Relative gene expression of the microglial activation marker *Iba1* in different brain regions. The data is expressed in fold change relative to WT mice fed with a normal diet. **e** Representative IF images of SN sections stained against IBA1 (in green). Scale bar, 150 μm. **f** IBA1 positive (IBA1+) area in different brain regions. WT, wildtype healthy control animals; TG, PLP-hαSyn mice. White bars: WT mice; gray bars: WT mice fed with HSD (TG + HSD); black bars: TG mice; red bars: TG mice fed with HSD (TG + HSD). Error bars indicate SEM. Two-way ANOVA: *p < 0.05, **p < 0.01, ***p < 0.001, ****p < 0.0001 (Bonferroni’s test)

To assess the effect of HSD on microglia we performed histological and molecular analyses for two different markers of microglial activation, IBA1 and CD68 [58, 59]. The increase of IBA1 and CD68 levels has been associated with α-syn accumulation and neurodegeneration in PD and MSA animal models [51, 53, 60–65]. In agreement with previous data [51], significant microglial activation was observed in PLP-hαSyn mouse brains compared to healthy controls (Fig. 1). Gene expression analysis showed upregulation of *Cd68mRNA* in the forebrain, midbrain and cerebellum of PLP-hαSyn mice (Fig. 1a). A significant upregulation of *Cd68mRNA* was also observed in the HSD PLP-hαSyn group compared to PLP-hαSyn mice fed with normal diet (Fig. 1a). However, immunohistological analysis only showed a significant increase of CD68 in PLP-hαSyn mice compared to healthy control animals with no specific effect of diet (Fig. 1b, c). Higher levels of CD68 were observed by immunofluorescence in striatum, substantia nigra (SN), pontine nuclei (PN), and cerebellar white matter (CBWM) of PLP-hαSyn animals compared to wildtypes with no effect of diet either in PLP-hαSyn or healthy control mice, keeping both high-salt groups similar levels to their normal diet groups (Fig. 1b, c). Similar results were obtained with IBA1 (Fig. 1d–f). A significant upregulation of *Iba1mRNA* was observed in the midbrain and cerebellum of PLP-hαSyn mice compared to wildtype animals (Fig. 1d). Immmunohistological analyses showed a significant increase of IBA1 levels in the SN, PN, and cerebellum of transgenic vs control mice (Fig. 1e, f). No effects of the diet were observed either in PLP-hαSyn or in healthy control animals discarding a specific effect of diet on microglial activation (Fig. 1e, f).

In order to assess the role of astroglia in the inflammatory response observed in PLP-hαSyn mice, we analyzed in the brain subregions the expression levels of *Gfap* (glial fibrillary acidic protein), a marker of astrogliosis. Significant upregulation of *Gfap mRNA* was only observed in the cerebellum of PLP-hαSyn with no effect of diet, discarding a general involvement of astroglia in neuroinflammation (Additional file 2: Figure S2).

### High-salt diet does not interfere with the neuroinflammatory signaling in the PLP-hαSyn brain

**Fig. 2:**
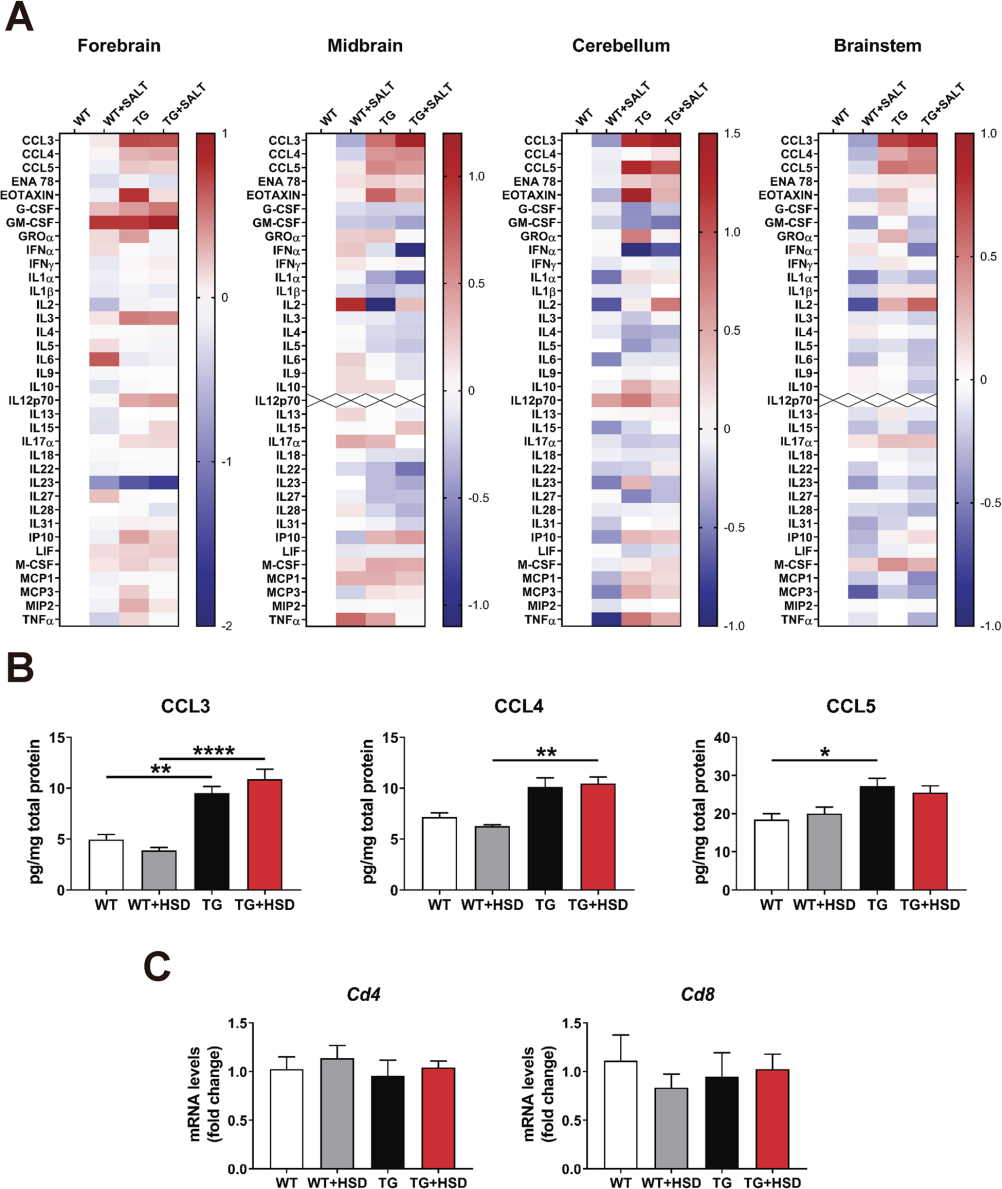
High-salt diet does not affect neuroinflammation in MSA mice. **a** Heat map comparing the log2 fold change in cytokine and chemokine expression in different brain regions of MSA mice fed with/without high-salt diet and control mice fed with high-salt, as referred to control mice fed with a normal diet. **b** Protein levels of CCL3, CCL4 and CCL5 chemokines in the midbrain. Protein levels are expressed in pg of protein of interest per mg of total proteins. **c** Relative gene expression of T-cell markers *Cd4* and *Cd8* in the midbrain. The data is expressed in fold change relative to WT mice fed with a normal diet. White bars: WT mice; gray bars: WT mice fed with HSD (TG + HSD); black bars: TG mice; red bars: TG mice fed with HSD (TG + HSD). Error bars indicate SEM. Two-way ANOVA: *p < 0.05, **p < 0.01, ****p < 0.0001 (Bonferroni’s test)

To further characterize the subregion-specific effect of HSD on neuroinflammation in PLP-α-syn mice, we evaluated separately the levels of 36 cytokines and chemokines in forebrain, midbrain, cerebellum, and brainstem by using a multi-analyte detection system (Fig. 2a, b). Heatmap portraying the overall changes of cytokines/chemokines in PLP-hαSyn and control mice showed different profiles between genotypes but no effect of diet (Fig.2a). The analysis revealed a significant increase of CCL3, CCL4, and CCL5 chemokines in PLP-hαSyn mouse brains compared to wildtype animals but no effect of salt (Fig. 2a-b and Additional file 3: Figure S3). The brain concentration of the remaining analytes showed no significant effect of genotype or diet (Additional file 4: Tables S1–S4).

We also evaluated changes in the adaptive immune system by analyzing the gene expression levels of two markers of T lymphocytes, *Cd4*, and *Cd8*. RT-qPCR analysis showed no changes between animal groups (Fig. 2c and Additional file 3: Figure S3), excluding an effect of the synucleinopathy or diet on T cell recruitment and discarding the involvement of the adaptive immune system in neuroinflammation in PLP-hαSyn mice (Fig. 2c and Additional file 3: Figure S3).

### High-salt diet causes no deterioration of motor impairment, neurodegeneration, and myelin dysfunction in PLP-hαSyn mice

**Fig. 3:**
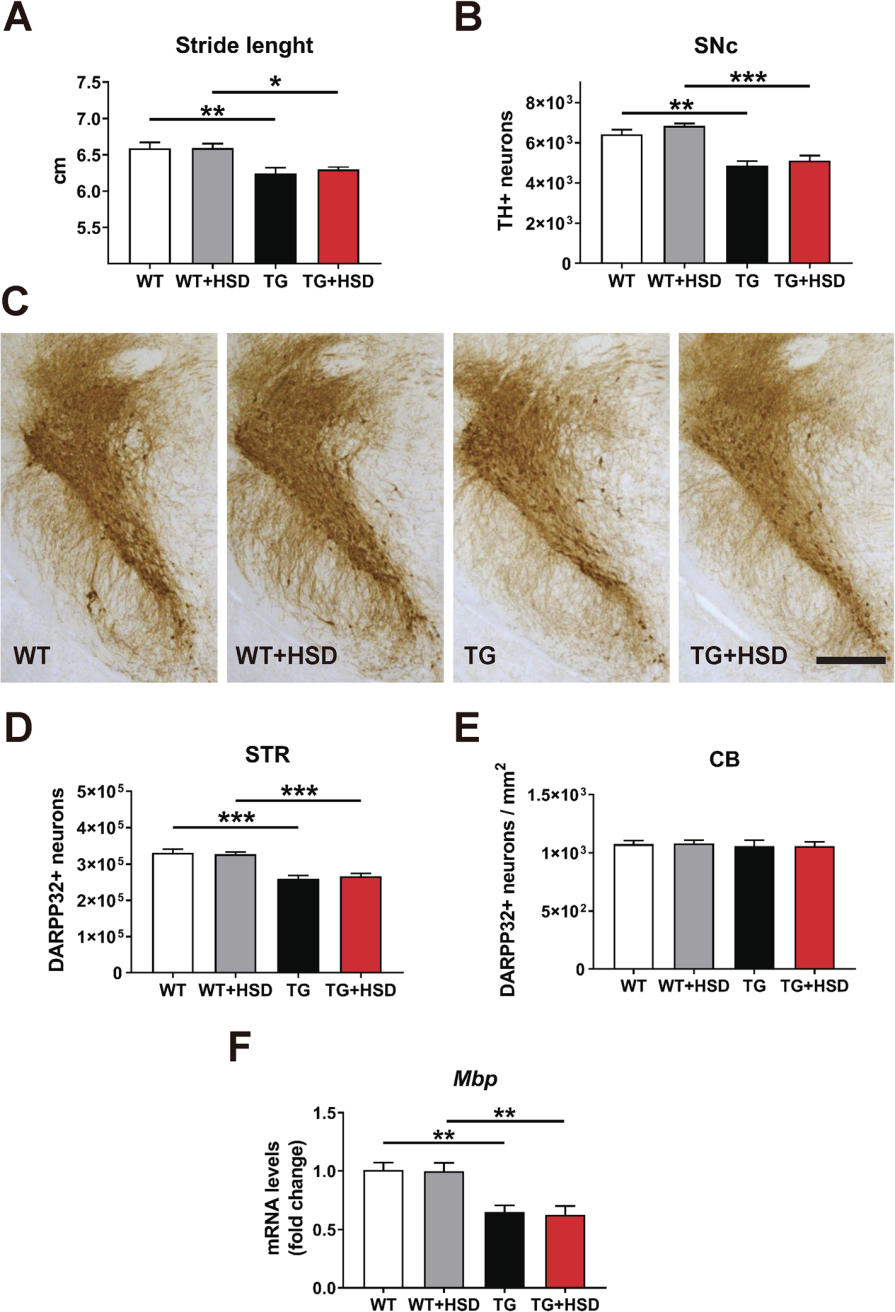
High-salt diet has no further harmful effect on motor impairment, neurodegeneration, and myelin dysfunction. **a** Gait analysis focused on stride length expressed in centimeter. **b** Stereological counting of the total number of dopaminergic (TH+) neurons in the entire substantia nigra pars compacta (SNc). **c** Representative images of SN sections stained against TH. Scale bar, 400 μm. **d** Stereological counting of the total number of medium spiny (DARPP32+) neurons in the entire STR. **e** Stereological counting of Purkinje (DARPP32+) neurons in the cerebellar cortex (CB). **f** Relative gene expression of *Mbp* in the midbrain. Error bars indicate SEM. Two-way ANOVA: *p < 0.05, **p < 0.01, ***p < 0.001 (Bonferroni’s test)

In order to evaluate the effect of diet on the gait impairment, we performed Digigait behavioral test. PLP-hαSyn mice showed a significant reduction of stride length compared to healthy control mice with no effect of diet on motor impairment (Fig. 3a). To assess the consequences of HSD on SND, the number of dopaminergic (TH+) neurons in the SNc and the number of MSNs (DARPP-32+) in the striatum were quantified in control and PLP-hαSyn mice. As previously described [51], stereological counting showed significant loss of TH+ and DARPP-32+ neurons in SNc and striatum respectively of PLP-hαSyn mice compared to wildtype animals (Fig. 3b–d). We did not detect any effect of diet neither in control nor in PLP-hαSyn mice (Fig. 3b–d). OPCA was evaluated by the stereological counting of Purkinje cells (DARPP32+) in the cerebellar cortex of PLP-hαSyn and control animals (Fig. 3e). No changes in the number of Purkinje neurons in the cerebellar cortex were observed between animal groups (Fig. 3e), discarding an expansion of the pathology after high-salt exposure similar to the ones observed previously after oxidative stress insult or proteasome inhibition [52, 56]. We also performed a subregion-specific evaluation of the effect of diet on myelin (Fig. 3f and Additional file 3: Figure S3). Gene expression analysis showed significant downregulation of *Mbp mRNA* (myelin basic protein) in PLP-hαSyn mice compared to healthy controls in most brain subregions (Fig. 3f and Additional file 3: Figure S3). However, we did not observe diet-associated differences neither in control nor in PLP-hαSyn animals, thus excluding a possible role of HSD in motor impairment, neurodegeneration, or myelination dysfunction (Fig. 3 and Additional file 3: Figure S3).

### High-salt diet does not affect α-syn pathology in PLP-hαSyn mice

**Fig. 4:**
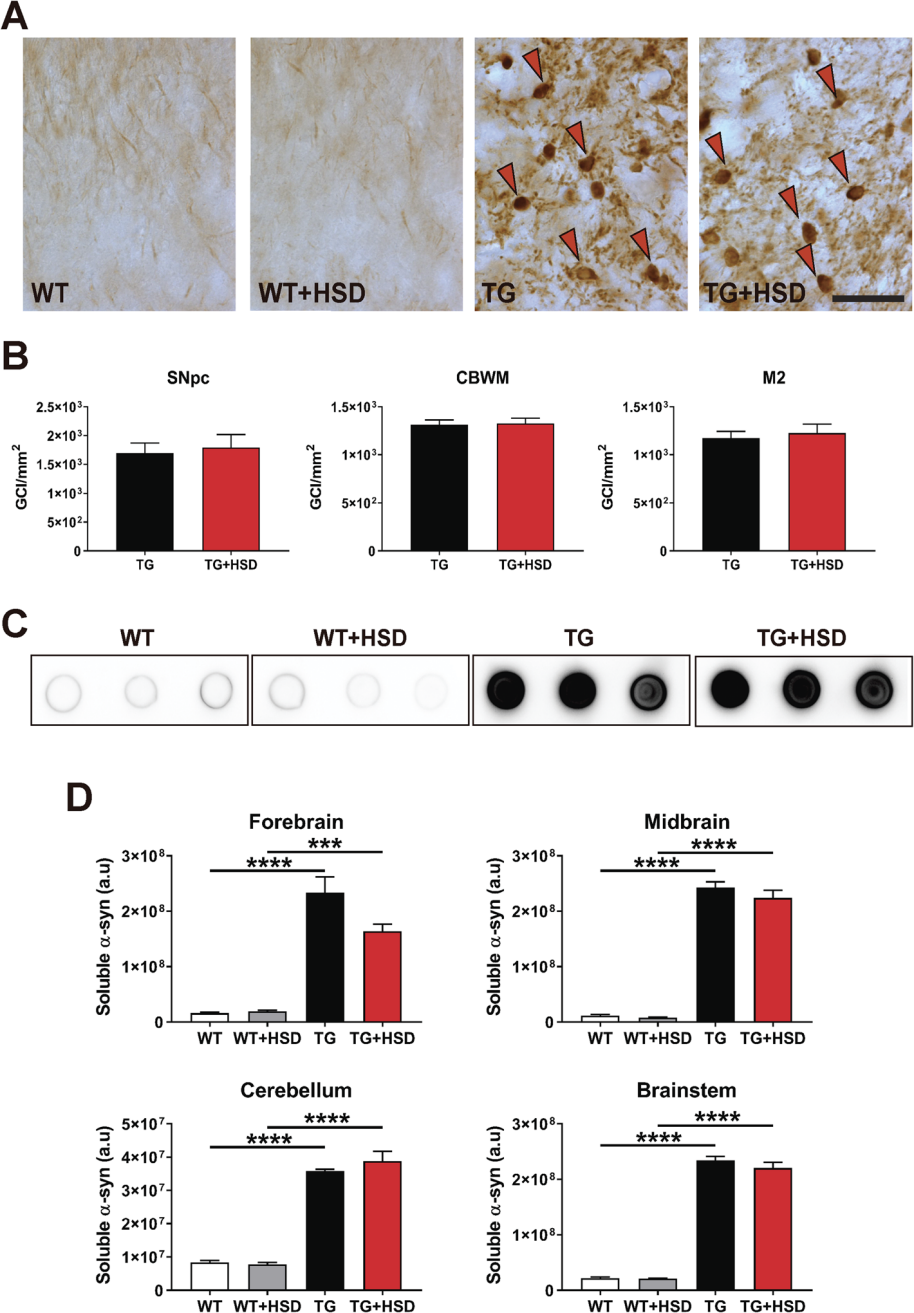
High-salt diet does not interfere with α-syn accumulation in MSA mice. **a** Representative images of SN sections stained against phosphorylated α-syn. Red arrows indicate individual GCI. Scale bar, 25 μm. **b** GCI density in SNc, CBWM, and motor cortex (M2) of PLP-hαSyn mice determined by stereological counting of brain sections stained against phosphorylated α-syn and expressed in GCI/mm^2^. **c** Representative images of DOT BLOT analysis for soluble human α-synuclein levels in the midbrain. **d** Quantification of soluble human α-synuclein levels in different brain areas. The data is shown in arbitrary units (a.u.). Error bars indicate SEM. Two-way ANOVA): ***p < 0.001, ****p < 0.0001 (Bonferroni’s test)

To fully evaluate the effect of HSD in the PLP-hαSyn mouse model, we assessed α-syn accumulation by histological and molecular analyses. In order to do that, representative brain sections of SNc, CBWM, and motor cortex (M2) were stained with anti-phosphorylated (p-S129) α-syn antibody and the density of GCIs was quantified (Fig. 4a, b). HSD showed no effect on GCI number in any of the regions analyzed (Fig. 4a, b). We also performed subregion-specific dot blot analysis of soluble α-syn (Fig. 4c, d). Significant higher levels of soluble α-syn were found in all brain subregions of PLP-hαSyn mice compared to wildtype animals, but no effect of diet was observed (Fig. 4c, d). Therefore, an effect of diet on α-syn accumulation was discarded.

## Discussion

Recently, several publications have associated HSD with activation of the immune system and neuroinflammation in experimental models of different neurological disorders, including MS [2–7, 11, 14, 16, 17, 66]. Despite the possible deleterious effect of salt on neuroinflammation, a high dietary intake of salt constitutes one of the most recommended non-pharmacological approaches to treat autonomic symptoms in MSA and PD patients [34]. Since microglial activation and neuroinflammation constitute two of the main pathological features of MSA and PD [24–26, 35–37], the use of HSD could aggravate CNS pathology by enhancing microglial activation, neuroinflammation, and the infiltration of peripheral immune cells.

In order to evaluate the effect of HSD on CNS pathology in α-synucleinopathies, PLP-hαSyn and wildtype animals were both fed with food pellets containing 0.19% (control diet) or 4% NaCl (high-salt diet). HSD consisting 4% NaCl constitutes an increase of about 8–19 times depending on the salt content in normal diet food pellets, which usually range between 0.19 and 0.4% depending on the study. For the human general population, the level of sodium intake recommended in major dietary guidelines ranges from 1200 to 2300 mg per day [67–70]. However, in MSA and PD, physicians often prescribe increased salt intake to around 10 g of salt per day [71–73], being 4–7 times higher than dietary recommendation for the general population. Thus, the experimental approach used in the present study is comparable to the spectrum of human salt consumption and may reflect changes due to HSD similar, or even stronger, than those expected in MSA and PD patients treated with salt. The deleterious effect of HSD in the CNS has also been described in rodents after exposure to 8% NaCl food pellets [11, 74]. However, this may not reflect physiological and clinically relevant conditions since it represents an increase of dietary salt intake of about 16–40 times compared to the normal diet.

**Fig. 5:**
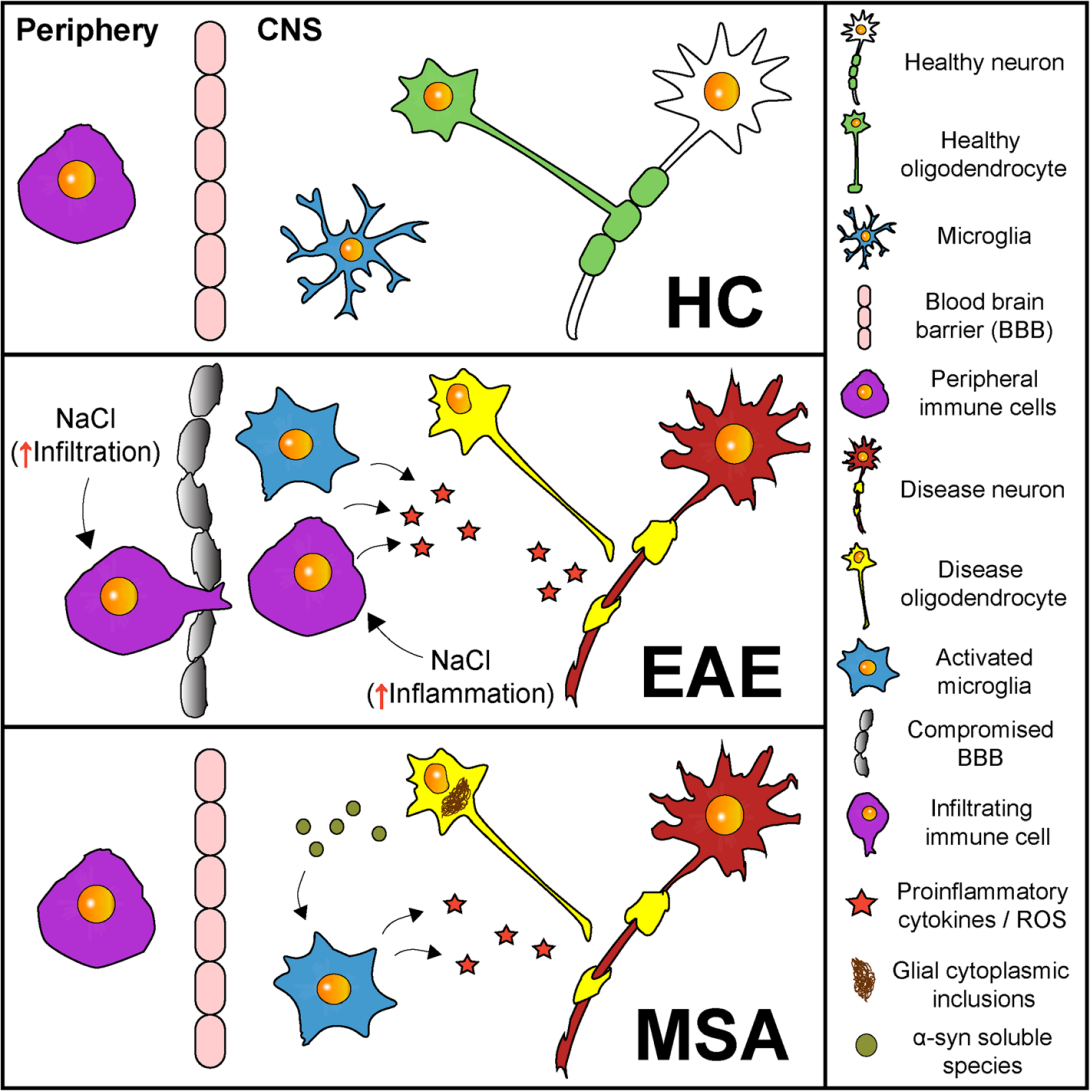
Pathophysiological features of MSA and EAE models and potential pathogenic effect of a high-salt diet. **a** Schematic overview of the central nervous system in healthy conditions. **b** In MSA, α-syn accumulates in the cytoplasm of oligodendrocytes inducing oligodendroglial dysfunction. Soluble α-syn oligomeric species spread through the brain parenchyma and trigger microglial activation and neuroinflammation. All these events lead to demyelination and neurodegeneration. High-salt diet exposure has no effect on the CNS of PLP-hαSyn mice possibly due to the absence of peripheral immune cell involvement in MSA brain pathology. **c** EAE mice model mimics many features of MS, such as blood-brain barrier (BBB) disruption, demyelinating lesions associated with infiltrating T cells, macrophages, and B cells, microglial activation, neuroinflammation, and neurodegeneration. In EAE mice, high-salt diet increases activation and infiltration of T cells and peripheral macrophages in the CNS accelerating disease onset, augmenting its severity and enhancing blood-brain barrier disruption and brain pathology

Here, we demonstrate that HSD does not affect the activation of microglial cells in PLP-hαSyn mice. Previous results from our group have shown a progressive increase in microglial activation with disease progression in this animal model [51] that can also be augmented in the presence of second deleterious stimuli such as mitochondrial dysfunction or proteasome impairment [52, 56]. In contrast, HSD did not increase microglial activation in the present study according to different molecular and histological analyses. We have also previously shown that the total number of microglial/macrophage cells (IBA1+) does not change in the CNS of MSA mice with disease progression and instead only an increase in their activation state is observed [51]. Therefore, the infiltration of peripheral macrophages in PLP-hαSyn mouse brains appears to be unlikely. Our results differ from experimental studies of inflammatory disorders associated with CNS infiltration of peripheral and monocyte-derived macrophages, where HSD increases activation and polarization towards an M1 phenotype [14, 16, 17] (Fig. 5). The difference between those studies and ours may reflect distinct cell-specific responses of peripheral macrophages and microglial cells to HSD due to their different developmental origin and activation patterns [75–78]. However, further analyses are required to fully understand these differences.

We also show that HSD does not interfere with neuroinflammation, astroglial activation, or T cell CNS infiltration in PLP-hαSyn mice. The absence of changes in different markers of T lymphocytes (CD4 and CD8) and their associated proinflammatory cytokines and chemokines (i.e., IFN-γ, IL-17, IL-12, IL-23) [13, 79–83] in the brain of MSA mice compared to wildtype animals suggest that these cells are not critical in the neuroinflammatory process observed in PLP-hαSyn mice. Moreover, human studies have shown no clear evidence of T cell involvement in MSA so far. The absence of a higher activation of the immune system in PLP-hαSyn mice could explain the differences with HSD studies in EAE models of MS where activation and infiltration of T cells and peripheral macrophages in the CNS accelerate disease onset, augment its severity, and enhance brain pathology [6–10, 12, 14, 15] (Fig. 5). However, the results obtained from our study cannot be extrapolated to PD, where an association between T cells and neurodegeneration has been recently suggested [38–43]. Further studies including pre-clinical models of PD are needed to clarify the effect of HSD on underlying neuropathology.

HSD did not affect neurodegeneration or demyelination in MSA mice. Although PLP-hαSyn animals develop SND and myelin dysfunction characterized respectively by a significant loss of dopaminergic neurons in the SNc and MSNs in the striatum and a downregulation of the *Mbp* gene in several brain subregions, no changes were observed after HSD exposure in these mice. These findings again differ from the studies in EAE models of MS where HSD has led to enhanced demyelination [14, 15]. In agreement with our data, a large clinical study has recently revealed that salt intake does not influence MS disease course or activity thus indicating that EAE models, which are induced by active immunization with myelin components or by passive transfer of autoreactive T cells, do not necessarily reflect the human disease [18, 84]. In contrast to the MSA model used in our study, where neuroinflammation is a secondary event induced by the aggregation of α-syn in oligodendrocytes [51, 53], neuroinflammation is a primary event induced by a peripheral immune response in the EAE models used for the studies on salt intake [9, 12, 13, 84]. Thus, the differences in the effects of diet on the neuropathology of MSA and MS animal models could also be explained by the absence of neuroinflammatory changes induced by HSD in PLP-hαSyn mice (Fig. 5).

Previous results from our group have shown that a combination of synucleinopathy with second hit stimuli such as oxidative stress or proteasome inhibition can aggravate the pathology in PLP-hαSyn mice towards full-blown MSA with strong microglial activation and spreading of SND, OPCA, and GCIs [52, 56]. However, PLP-hαSyn mice showed no changes in α-syn accumulation after HSD exposure by neither molecular nor histological analyses, therefore excluding an effect of diet on any of the synucleinopathy features.

In contrast to our observations, two recent studies by Faraco et al. have shown that HSD induces cognitive impairment in wildtype animals [11, 74]. Faraco et al. observed significant deleterious effects in wildtype mice after 12 weeks of HSD with 4% NaCl food pellets, a similar protocol to the one used in our study. However, we did not find differences between wildtype animals fed with normal or HSD. These differences may be explained by the use of different mouse substrains. The PLP-hαSyn and control animals used in our study were C57BL/6 N. Unfortunately, Faraco et al. do not mention which specific C57BL/6 substrain was used in their study. It has been shown that C57BL/6 substrains present behavioral [85] and genetic differences—including immune function —[86]. Moreover, other groups have shown that HSD has either no effect or even ameliorates symptoms in animal models of other inflammatory disorders [87, 88]. Thus, the differences between our data and previous HSD studies, and in particular data in EAE mice, may be explained by the use of different mouse strains or by the different experimental approaches used to boost the inflammatory process, as previously discussed.

Despite the publications supporting the deleterious effect of HSD in experimental models of MS, two large clinical studies failed to show an association between salt intake and higher MS disease risk, progression, or activity [18, 19]. Moreover, although sodium intake differs between East Asian, American, and European population [89], MSA cohort study groups from Japan, Europe, and the USA show a similar median survival [90–92], supporting the current findings and suggesting that salt consumption differences may not affect disease progression in MSA.

Several limitations of the current study need to be acknowledged. Despite not having observed an effect of diet in the CNS of MSA mice, we cannot exclude an effect in peripheral organs. Moreover, in the present study, we have not evaluated the effect of HSD on cardiovascular regulation or pathology. Among the different autonomic cardiovascular problems present in MSA patients, the presence of orthostatic hypotension (OH) constitutes one of the major criteria for diagnosis [93]. Data from our group show cardiovascular defects in PLP-hαSyn mice [50], but it is not possible to address the issue of OH in a mouse model. In this regard, we cannot exclude a role of high sodium intake on neuropathology linked to OH in MSA patients. However, the analysis of the effect of diet in both the peripheral immune system and the different cardiovascular features of PLP-hαSyn mice is beyond the scope of the present study, where the main objective was to study the effect of HSD in the neuroinflammatory process underlying MSA pathology in the CNS.

## Conclusions

Here, we demonstrate that HSD does not interfere with microglial activation, neuroinflammation, motor function, neurodegeneration, and α-syn accumulation in the PLP-hαSyn mouse model of MSA, making deleterious effects of HSD on brain pathology and its progression unlikely. Our findings contrast with experimental data obtained in EAE models of MS and do not support a pro-neuroinflammatory effect of the current clinical practice of a high-salt diet for the treatment of autonomic failure in MSA.

## Supplementary information

**Additional file 1: Figure S1**. *Animal body weight*. Body weight of healthy control mice fed with normal diet (WT; white circles) or HSD (WT + HSD; gray circles) and PLP-hαSyn mice fed with normal diet (TG; black squares) or HSD (TG + HSD; red squares). Body weight was measured weekly throughout the treatment period.

**Additional file 2: Figure S2**. *High-salt diet does not affect astrogliosis in MSA mice*. Gene expression (mRNA) levels in forebrain, midbrain, cerebellum and brainstem of the astrocyte marker *Gfap*. mRNA levels are expressed in fold change relative to WT mice fed with normal diet. White bars: WT mice; Gray bars: WT mice fed with HSD (TG + HSD); Black bars: TG mice; Red bars: TG mice fed with HSD (TG + HSD). Error bars indicate SEM. Two-way ANOVA: *** *p* < 0.001, **** *p* < 0.0001 (Bonferroni’s test).

**Additional file 3: Figure S3**. *Protein levels and gene expression (mRNA) levels in forebrain, cerebellum and brainstem of the main proteins and genes analyzed on the study*. Protein levels are expressed in pg of protein of interest per mg of total proteins. mRNA levels are expressed in fold change relative to WT mice fed with normal diet. White bars: WT mice; Gray bars: WT mice fed with HSD (TG + HSD); Black bars: TG mice; Red bars: TG mice fed with HSD (TG + HSD). Error bars indicate SEM. Two-way ANOVA: * *p* < 0.05, ** *p* < 0.01, *** *p* < 0.001 (Bonferroni’s test).

**Additional file 4: Tables S1**. *Cytokine and chemokine protein levels in forebrain*. The table represent the concentrations (pg/mg total protein) of the different cytokines and chemokines measured in forebrain of control and MSA mice fed with normal or high salt diet. Data are presented as mean ± SD. *p*-values where obtained by two-way ANOVA with Bonferroni’s post hoc test. **Tables S2**. *Cytokine and chemokine protein levels in midbrain*. The table represent the concentrations (pg/mg total protein) of the different cytokines and chemokines measured in forebrain of control and MSA mice fed with normal or high salt diet. Data are presented as mean ± SD. p-values where obtained by two-way ANOVA with Bonferroni’s post hoc test. **Tables S3**. *Cytokine and chemokine protein levels in cerebellum*. The table represent the concentrations (pg/mg total protein) of the different cytokines and chemokines measured in forebrain of control and MSA mice fed with normal or high salt diet. Data are presented as mean ± SD. *p*-values where obtained by two-way ANOVA with Bonferroni’s post hoc test. **Tables S4**. *Cytokine and chemokine protein levels in brainstem*. The table represent the concentrations (pg/mg total protein) of the different cytokines and chemokines measured in forebrain of control and MSA mice fed with normal or high salt diet. Data are presented as mean ± SD. *p*-values where obtained by two-way ANOVA with Bonferroni’s post hoc test.

## Data Availability

The data that support the findings of this study are available from the corresponding author upon reasonable request.

## Abbreviations

CBWM: Cerebellar white matter
CNS: Central nervous system
EAE: Experimental autoimmune encephalomyelitis
GCIs: Glial cytoplasmic inclusions
HSD: High-salt diet
MS: Multiple sclerosis
MSA: Multiple system atrophy
MSNs: Medium spiny neurons
nOH: Neurogenic orthostatic hypotension
OPCA: Olivopontocerebellar atrophy
PD: Parkinson’s disease
PN: Pontine nuclei
SN: Substantia nigra
SNc: Substantia nigra pars compacta
SND: Striatonigral degeneration
TH: Tyrosine hydroxylase
α-syn: Alpha-synuclein
