# Supplementary figures and images for: High-salt diet does not boost neuroinflammation and neurodegeneration in a model of α-synucleinopathy
Source: J Neuroinflammation. 2020 Jan 24;17:35. doi: 10.1186/s12974-020-1714-y (PMC6982394; doi:10.1186/s12974-020-1714-y)

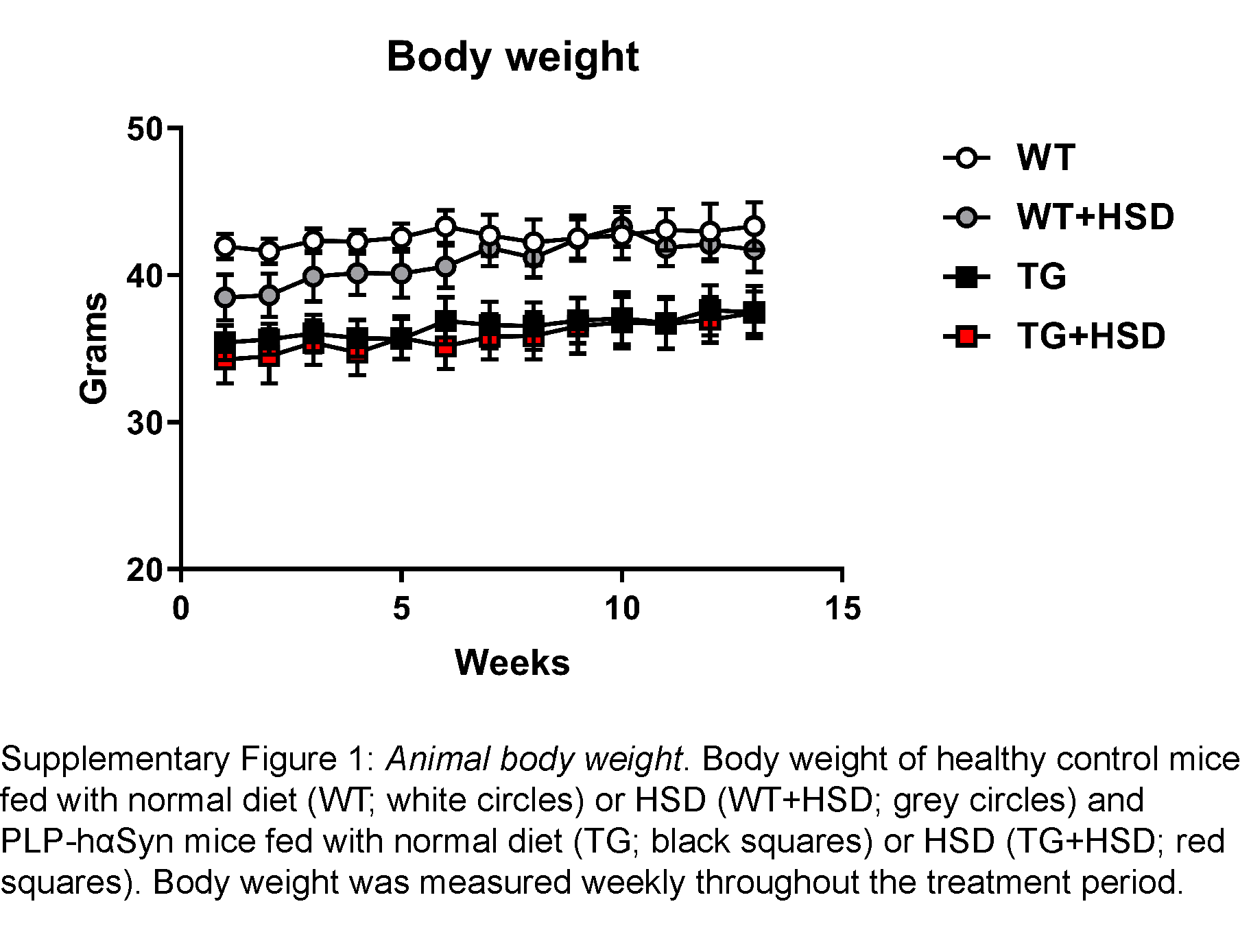

Supplement: Supplementary file 1 — Additional file 1: Figure S1. Animal body weight. Body weight of healthy control mice fed with normal diet (WT; white circles) or HSD (WT + HSD; gray circles) and PLP-hαSyn mice fed with normal diet (TG; black squares) or HSD (TG + HSD; red squares). Body weight was measured weekly throughout the treatment period. [file 12974_2020_1714_MOESM1_ESM.tif]

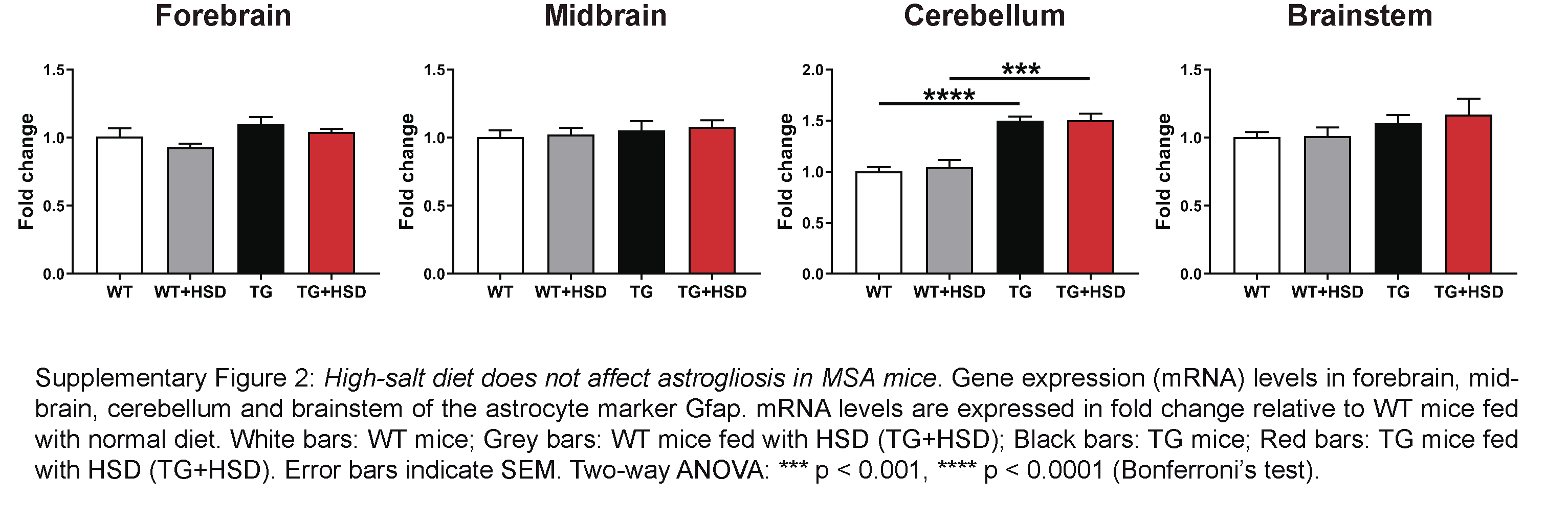

Supplement: Supplementary file 2 — Additional file 2: Figure S2. High-salt diet does not affect astrogliosis in MSA mice. Gene expression (mRNA) levels in forebrain, midbrain, cerebellum and brainstem of the astrocyte marker Gfap. mRNA levels are expressed in fold change relative to WT mice fed with normal diet. White bars: WT mice; Gray bars: WT mice fed with HSD (TG + HSD); Black bars: TG mice; Red bars: TG mice fed with HSD (TG + HSD). Error bars indicate SEM. Two-way ANOVA: *** p < 0.001, **** p < 0.0001 (Bonferroni’s test). [file 12974_2020_1714_MOESM2_ESM.tif]

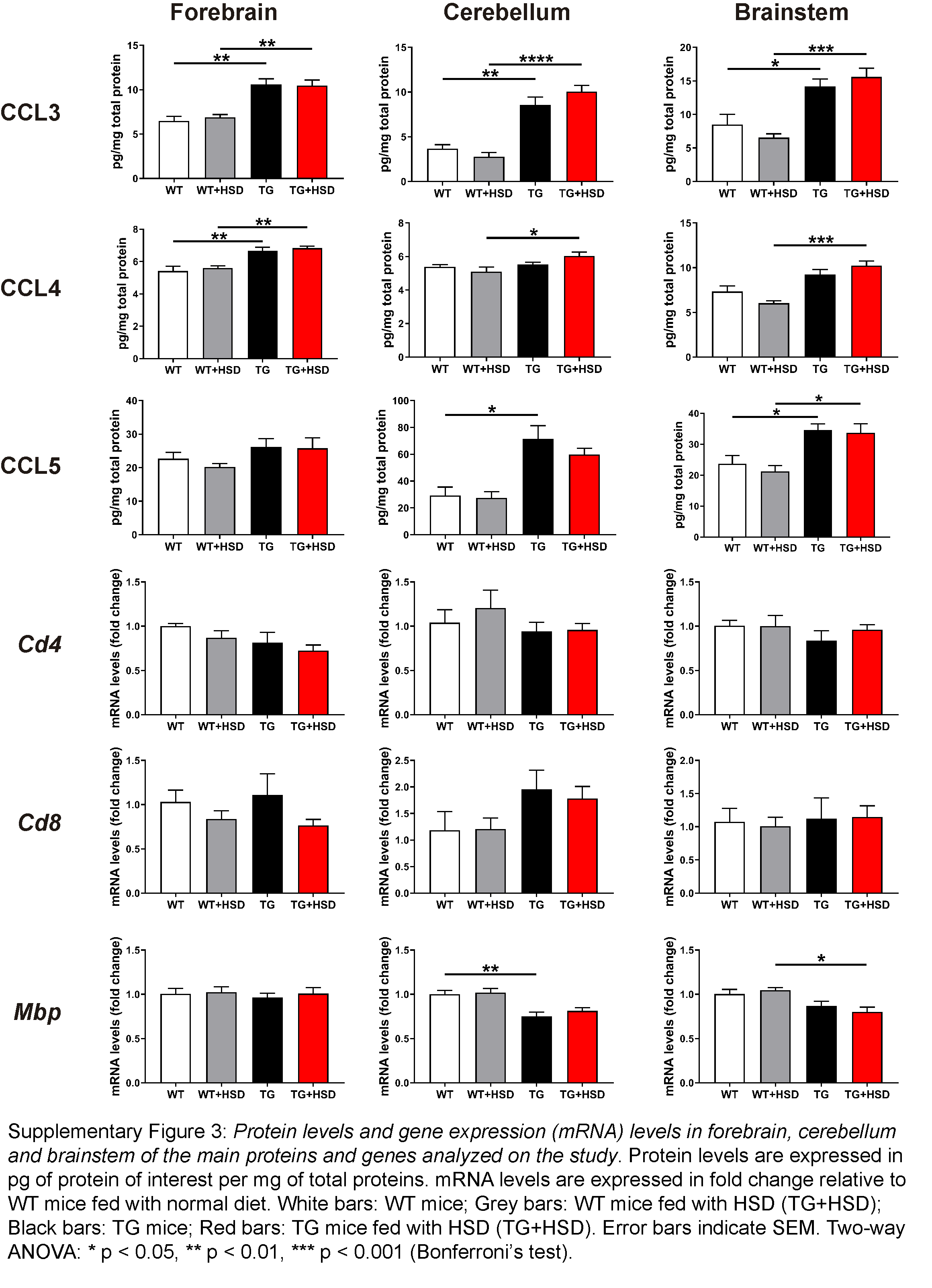

Supplement: Supplementary file 3 — Additional file 3: Figure S3. Protein levels and gene expression (mRNA) levels in forebrain, cerebellum and brainstem of the main proteins and genes analyzed on the study. Protein levels are expressed in pg of protein of interest per mg of total proteins. mRNA levels are expressed in fold change relative to WT mice fed with normal diet. White bars: WT mice; Gray bars: WT mice fed with HSD (TG + HSD); Black bars: TG mice; Red bars: TG mice fed with HSD (TG + HSD). Error bars indicate SEM. Two-way ANOVA: * p < 0.05, ** p < 0.01, *** p < 0.001 (Bonferroni’s test). [file 12974_2020_1714_MOESM3_ESM.tif]
